# Bone marrow angiogenesis and mast cell density increase simultaneously with progression of human multiple myeloma

**DOI:** 10.1038/sj.bjc.6690070

**Published:** 1999-02

**Authors:** D Ribatti, A Vacca, B Nico, F Quondamatteo, R Ria, M Minischetti, A Marzullo, R Herken, L Roncali, F Dammacco

**Affiliations:** Institute of Human Anatomy, Histology and Embryology, University of Bari Medical School, Bari, I-70124, Italy; Department of Biomedical Sciences and Human Oncology, University of Bari Medical School, Bari, I-70124, Italy; Zentrum Anatomie Abteilung Histologie, Universitat Gottingen, Gottingen, D-37075, Germany; Institute of Pathology, University of Bari Medical School, Bari, I-70124, Italy

**Keywords:** angiogenesis, mast cell, multiple myeloma, tumour progression

## Abstract

Immunohistochemical, cytochemical and ultrastructural data showing vivid angiogenesis and numerous mast cells (MCs) in the bone marrow of 24 patients with active multiple myeloma (MM) compared with 34 patients with non-active MM and 22 patients with monoclonal gammopathy of undetermined significance (MGUS) led us to hypothesize that angiogenesis parallels progression of MM, and that MCs participate in its induction via angiogenic factors in their secretory granules. © 1999 Cancer Research Campaign


*British Journal of Cance* (1999) **79**(3/4), 451–455
© 1999 Cancer Research Campaign
Article no. bjoc.1998.0070

### Bone marrow angiogenesis and mast cell density increase simultaneously with progression of human multiple myeloma

**D Ribatti[1], A Vacca[2], B Nico[1], F Quondamatteo[3], R Ria[2], M Minischetti[2], A Marzullo[4], R Herken[3], L Roncali[1] and F Dammacco[2]**

[1]Institute of Human Anatomy, Histology and Embryology; [2]Department of Biomedical Sciences and Human Oncology, University of Bari Medical School, I-70124 Bari, Italy; [3]Zentrum Anatomie Abteilung Histologie, Universitat Gottingen, D-37075 Gottingen, Germany; [4]Institute of Pathology, University of Bari Medical School, I-70124 Bari, Italy

**Summary** Immunohistochemical, cytochemical and ultrastructural data showing vivid angiogenesis and numerous mast cells (MCs) in the bone marrow of 24 patients with active multiple myeloma (MM) compared with 34 patients with non-active MM and 22 patients with monoclonal gammopathy of undetermined significance (MGUS) led us to hypothesize that angiogenesis parallels progression of MM, and that MCs participate in its induction via angiogenic factors in their secretory granules.

**Keywords:** angiogenesis; mast cell; multiple myeloma; tumour progression

Angiogenesis is obligatory in the enhancement of progression (growth, invasion and metastasis) of solid tumours (Folkman, 1995). New vessels promote growth by conveying oxygen and nutrients and removing catabolites, whereas endothelial cells secrete growth factors for tumour cells (Hamada et al, 1992; Folkman, 1995). Endothelial cells also secrete a variety of matrix-degrading proteinases which facilitate invasion (Mignatti and Rifkin, 1993). Lastly, an expanding endothelial surface increases opportunities for tumour cells to enter the circulation and metastasize (Aznavoorian et al, 1993).

Tumour cells may not be the only source of angiogenic factors within a tumour. Host inflammatory cells, including fibroblasts, macrophages and mast cells (MCs), which are recruited and activated by tumour cells via paracrine mechanisms act synergically with these cells by secreting the same or other factors (Polverini, 1996). MCs play a decisive role in the synergism (Norrby and Whooley, 1993). Also, experimentally induced tumours display MC accumulation close to the tumour cells before the onset of angiogenesis (Kessler et al, 1976), and those induced in MC-deficient mice display both reduced angiogenesis and ability to metastasize (Starkey et al, 1988; Dethlefsen et al, 1994).

Knowledge on these relations in haematological tumours is circumstantial. Angiogenesis is correlated with tumour growth (S-phase fraction) in monoclonal gammopathies (Vacca et al, 1994), and with progression stages in B-cell non-Hodgkin's lymphomas (Ribatti et al, 1998) and in mycosis fungoides (Vacca et al, 1997).

This paper presents the results of an investigation on angiogenesis and MC counts in the bone marrow of patients with monoclonal gammopathy of undetermined significance (MGUS) and multiple myeloma (MM) grouped according to a pathway of progression.

*Received 16 March 1998*
*Revised 28 May 1998*
*Accepted 3 June 1998*

*Correspondence to:* D Ribatti, Institute of Human Anatomy, Piazza G. Cesare 11, Policlinico, I-70124 Bari, Italy

### MATERIALS AND METHODS

### Patients

A total of 80 Caucasian patients who fulfilled the South West Oncology Group diagnostic criteria for MM and MGUS (Durie, 1991) were studied (Table 1). Myeloma patients were defined as active or non-active, according to clinical performance and

**Table 1** Patient clinical and immunological data

| | |
|---|---|
| Total no. | 80 |
| Multiple myeloma | 58 |
| Active | 24 |
| Average age (median, range) | 64 years (66.5, 42–87) |
| Men/women | 15/9 |
| M-component IgG/IgA/IgD κ/or λ | 16/6/1/1 |
| Diagnosis | 10 |
| Stage I/II/III; A/B[a] | 1/2/7; 6/4 |
| Relapse[b] | 8 |
| Progression | 6 |
| Non-active | 34 |
| Average age (median, range) | 66 years (68, 45–80) |
| Men/women | 20/14 |
| M-component IgG/IgA κ or λ | 22/8/4 |
| Response | 20 |
| Plateau[c] | 14 |
| Monoclonal gammopathy of undetermined significance | 22 |
| Average age (median, range) | 62 years (63.8, 45–86) |
| Men/women | 12/10 |
| M-component IgG/IgA/IgM | 18/2/2 |

[a]According to Durie and Salmon. [b]Relapse defined as M-component increase >50% from the lowest value, or clinical and bone marrow relapse when the M-component did not reflect tumour load and disease activity. [c]Plateau phase defined as post-treatment M-component decrease >50%, and lasting for at least 6 months without treatment.

**Table 2** Microvessel area and mast cell counts in the bone marrow of patients

| | MGUS ($n$ = 22) | Non-active MM ($n$ = 34) | Active MM ($n$ = 24) |
|---|---|---|---|
| Microvessel area (μm²) | 1.1 ± 0.5 (0.9; 0.2–2.5) | 1.2 ± 0.6 (1.3; 0.2–2.2) | 5.7 ± 3* (5.2; 1.2–12.8) |
| Number of mast cells | 1.3 ± 1* (1; 0–3) | 1.6 ± 1.2 (1.5; 0–4) | 4.8 ± 2* (5; 1–8) |

Results are expressed as means ± 1 standard deviation (median; range) in 250× microscopic fields (125 μm²). The cellular area in MGUS, non-active and active MM was 42.1 ± 8.8 μm², 46.6 ± 11.2 μm² and 52.4 ± 9.6 μm². *$P$ < 0.01 compared with non-active MM and MGUS

M-component level (Durie, 1991). Active patients were those: (a) at diagnosis, with symptomatic disease and an increase in the M-component level in the 3 months before analysis; (b) at relapse; (c) with unresponsive and rapidly progressive disease (leukaemic progression), characterized by severe bone pain, hypercalcaemia and pancytopenia. Non-active patients were those in: (a) post-treatment complete/objective response; (b) the off-treatment plateau phase. MGUS, non-active-MM and active MM constitute a progression pathway because: (i) the clinical evolution from one step to the next is typical; (ii) the plasma cell S-phase fraction and tumour mass rise significantly in the transition from one step to the next (Durie, 1991).

The study was approved by the local ethics committee and all patients gave their informed consent.

### Measurement of bone marrow angiogenesis

All blood vessels were displayed in 6-μm sections of 4% paraformaldehyde-fixed paraffin-embedded biopsies by staining endothelial cells with the anti-factor VIII murine monoclonal antibody M616 (IgG1; Dako, Glostrup, Denmark) and a three-layer biotin–avidin–peroxidase system described previously (Vacca et al, 1994). The very few megakaryocytes also stained by factor VIII

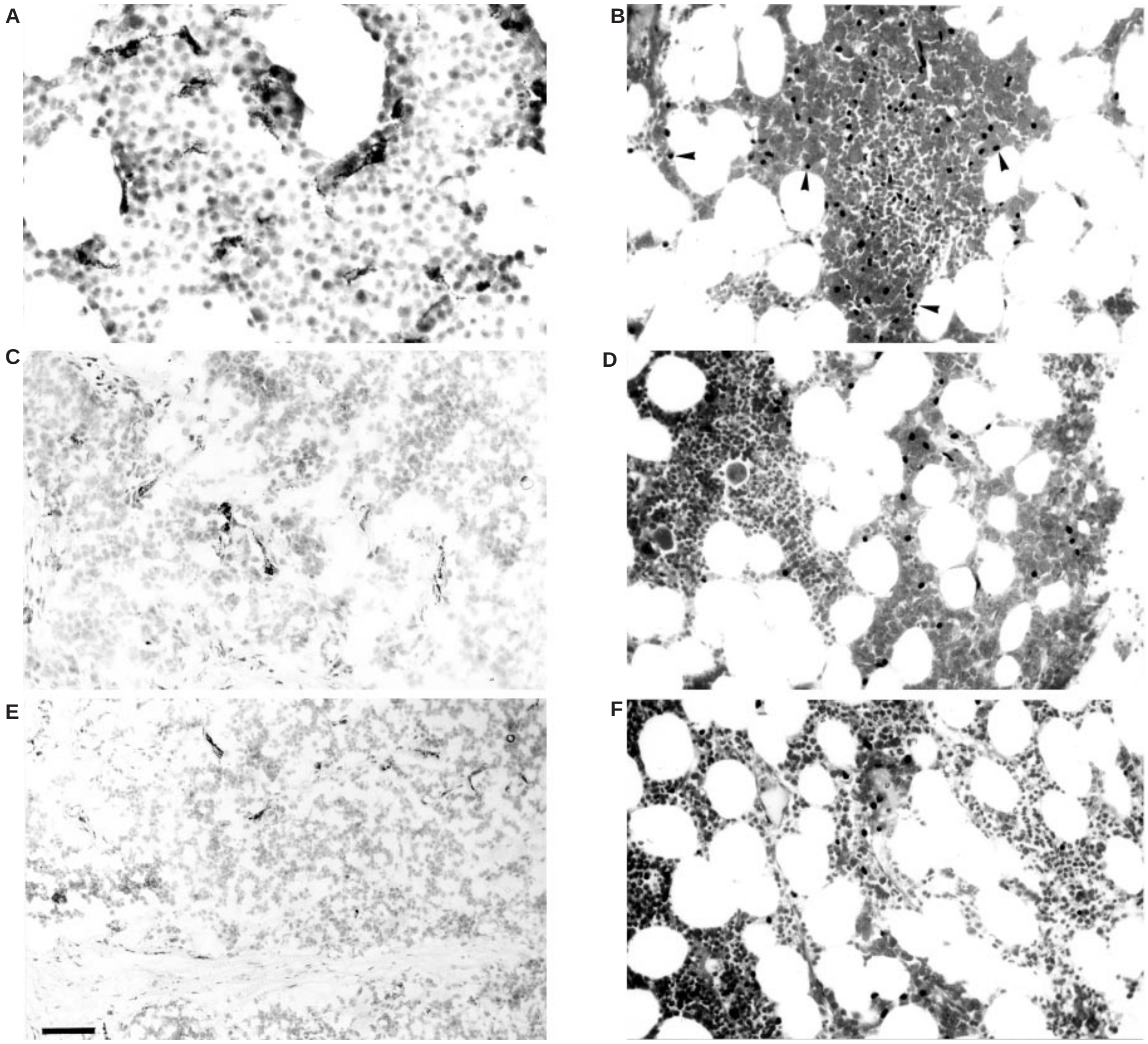

**Figure 1** Adjacent sections of bone marrow biopsies stained with factor VIII for microvessels (**A**, **C**, **E**) and with toluidine blue for mast cells (**B**, **D**, **F**) from patients with: (**A**) and (**B**) active MM (relapse); (**C**) and (**D**) non-active MM (plateau); and (**E**) and (**F**) MGUS. Note the higher density of microvessels and mast cells (some are arrowheaded) in the active MM patient. Bar = 10 μm

 © Cancer Research Campaign 1999

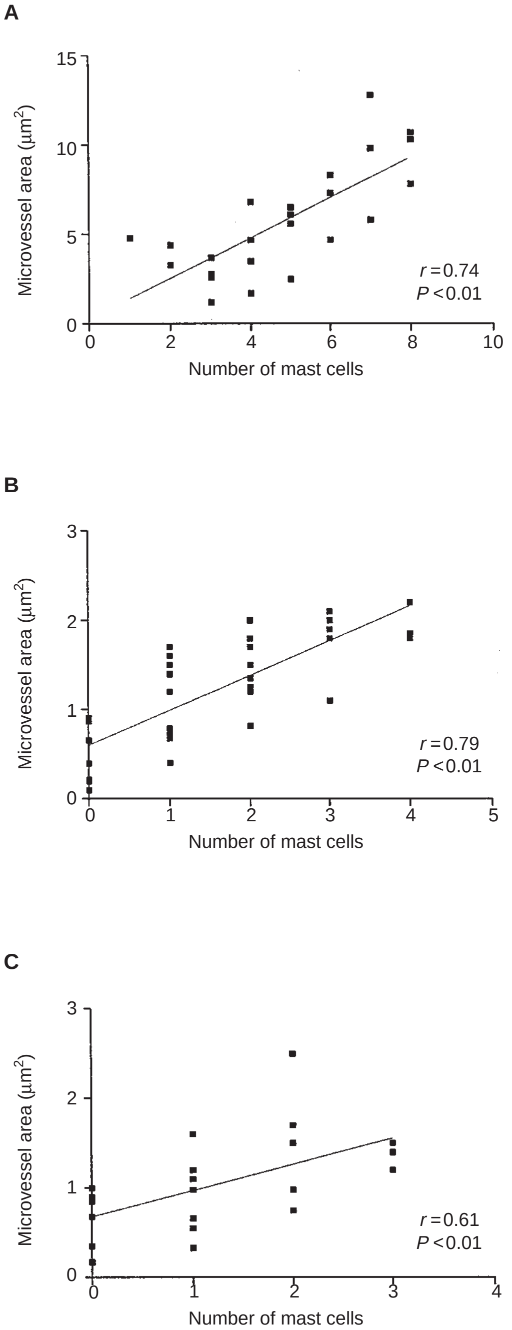

**Figure 2** Mast cell counts in comparison with the microvessel area in the bone marrow of patients with (**A**) active and (**B**) non-active MM and (**C**) with MGUS. Significance of the regression analysis was calculated by the Pearson's (*r*) test

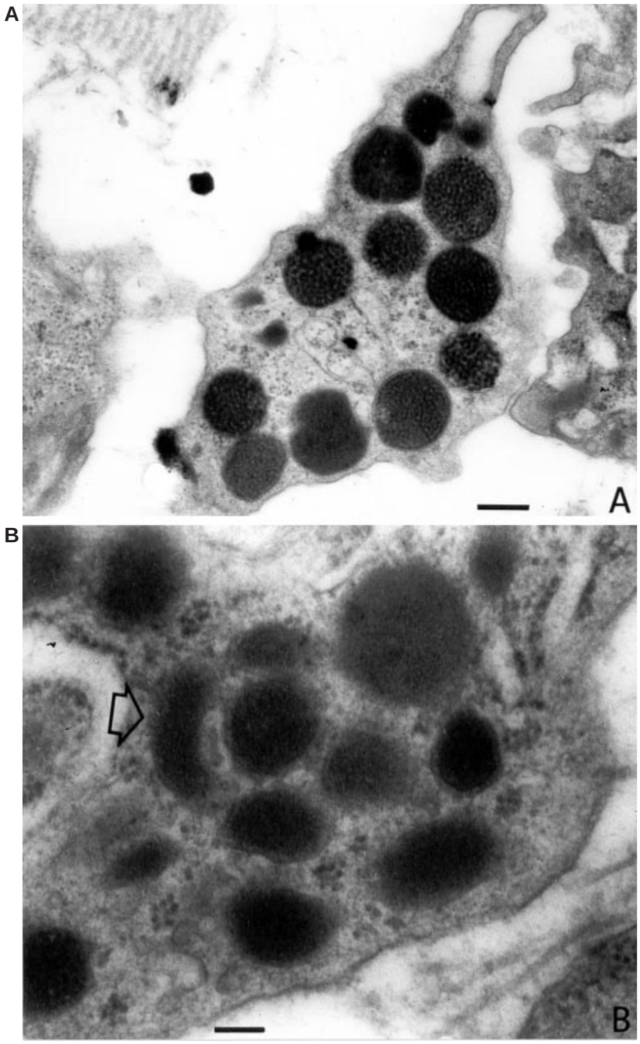

**Figure 3** Ultrastructural findings of bone marrow biopsies from patients with active MM. In (**A**), a mast cell with typical electron-dense round granules and in (**B**), at higher magnification, a cytoplasmic granule with a semilunar aspect (arrow), among other typical round granules, is recognizable. Bar, (**A**), 0.08 μm; (**B**) 0.02 μm were easily distinguishable by their morphology. Angiogenesis was measured as microvessel area without knowledge of final diagnosis. Briefly, six to eight 250× fields covering the whole of each of two sections per biopsy were examined with a superimposed 484-point square reticulum (125 $\mu m^2$) to identify microvessels (capillaries and small venules) as endothelial cells either single or clustered in nests or tubes, and clearly separated from one another, and either without or with a lumen (not exceeding 10 μm). A planimetric point count method (Elias and Hyde, 1983) with slight modifications for the computed image analysis (Leica Quantimet 500, Wetzlar, Germany) was applied to measure the microvessel area within the cellular area (reticulum area minus connective tissue, fat, bone lamellae, necrosis and haemorrhage areas) (Vacca et al, 1994). Values are expressed as means ± 1 standard deviation (s.d.) per group of patients.

### MC counts

MCs were highlighted in two sections adjacent to that stained for microvessels with 0.5% aqueous solution of toluidine blue (Merk, Darmstadt, Germany). Cells were counted in six to eight 250× fields inside the reticulum and calculated as means ± 1 s.d. for each group of patients.

### Electron microscopy

Small pieces (approximately 1 $mm^3$) of tissue were fixed in 3% glutaraldehyde in 0.1 M phosphate-buffered saline (PBS) for 3 h,

© Cancer Research Campaign 1999

washed in the same buffer for 12 h, post-fixed in 1% osmium tetroxide, dehydrated in graded ethanols and embedded in Epon 812. Ultrathin sections were cut with a diamond knife on a LKB V ultratome, stained with uranyl acetate followed by lead citrate, and examined in a 9A Zeiss electron microscope.

### Statistics

The significance of changes in the microvessel area and MC counts in the groups was determined with the parametric (Fisher's test) and non-parametric (Kruskal–Wallis test) analysis of variance, followed by Duncan (*t*), Bonferroni (*t*), and Wilcoxon tests to compare groups two by two. Correlations between microvessel area and MC counts in the groups were assessed with the Pearson's (r) coefficient and simple regression analysis. Data were computed with the Statistical Analysis Software (SAS, SAS Institute, Cary, NC, USA).

### RESULTS

Table 2 shows the microvessel area normalized to the total cellular area and the MC counts on bone marrow adjacent sections of patients with active MM, non-active MM and MGUS. The area was significantly larger in patients with active MM than in those with non-active MM and with MGUS, between which variations were negligible. In parallel, the MC counts were significantly higher in active MM than in the other groups. The differences in microvessels and MC are also shown in Figure 1. The within-group comparison showed that both parameters were always significantly correlated (Figure 2). At the ultrastructural level, typical MCs with cytoplasmic matrix filled by numerous electron dense secretory granules (Figure 3A), and MCs with semilunar aspect of granules (Figure 3B) were recognizable. The latter imply slow, chronic release of mediators in response to a moderate, progressive, degranulatory stimulus (Kops et al, 1984; Ribatti et al, 1988).

### DISCUSSION

In the current study, we showed that bone marrow angiogenesis (evaluated as microvessel area) and MC counts were highly correlated in patients with non-active and active MM and in those with MGUS, and that both parameters increased simultaneously in active MM. As the progression from in situ to invasive and metastatic solid tumours is accompained and facilitated by the switch from the prevascular to the vascular phase (Hanahan and Folkman, 1996), our findings suggest that the active MM represent the 'vascular phase' of plasma cell tumours, whereas the non-active MM and MGUS represent the 'prevascular phase'. Bone marrow angiogenesis may, therefore, favour the progression from MGUS or non-active MM to active MM. As in solid tumours, where angiogenesis could be stimulated either directly or indirectly, after the tumour cells have recruited inflammatory cells stimulating them to secrete their own angiogenic factors (Hanahan and Folkman, 1996), the switch in MGUS and non-active MM may be induced by tumour plasma cells via secretion of angiogenic factors, namely interleukin 1 (IL-1) (Cozzolino et al, 1989), IL-6 (Schwab et al, 1991), tumour necrosis factor alpha (TNF-$\alpha$) (Lichtenstein et al, 1989), macrophage colony-stimulating factor (M-CSF) (Nakamura et al, 1989), transforming growth factor beta (TGF-$\beta$) (Klein, 1995), and by inflammatory cells, including MCs, via secretion of their angiogenic factors.

MCs are strikingly associated with angiogenesis, as found in chronic inflammatory diseases, namely rheumatoid arthritis and psoriasis, and in tumours, namely haemangiomas, carcinomas and lymphomas (Meininger and Zetter, 1992; Norrby and Woolley, 1993; Qu et al, 1995; Ribatti et al, 1998). In tumours, MCs are recruited and activated via several factors secreted by tumour cells: the c-*kit* receptor, or stem cells factor (Poole and Zetter, 1983; Norrby and Wooley, 1993), as well as the basic fibroblast growth factor (FGF-2), vascular endothelial growth factor (VEGF-A) and platelet-derived endothelial cell growth factor (Gruber et al, 1995). MCs contain, in secretory granules, heparin that in vitro stimulates endothelial cell proliferation and migration (Thorton et al, 1983; Alessandri et al, 1984), whereas in vivo it has been shown to have variable effects on angiogenesis; it may, thus, stimulate (Ribatti et al, 1987; Norrby, 1993), inhibit (Jakobson and Hahnenberger, 1991; Norrby, 1993; Wilks et al, 1991) or have no effect (Castellot et al, 1982; Taylor and Folkman, 1982). Histamine and tryptase, other MC-derived factors, also stimulate angiogenesis (Sorbo et al, 1994; Blair et al, 1997). In addition, MCs produce a variety of multifunctional cytokines and growth factors, such as IL-6 and IL-8 (Motro et al, 1990; Norrby, 1996), TNF-$\alpha$ (Beil et al, 1994), granulocyte–macrophage colony-stimulating factor (GM-CSF) (Bussolino et al, 1991), TGF-$\beta$ (Roberts et al, 1986), FGF-2 (Qu et al, 1995) and VEGF-A (Grutzkan et al, 1996), which may contribute to angiogenesis in active MM.

As concerns the ultrastructural features of MCs, the semilunar, or partial degranulating, aspect of their secretory granules, unlike IgE-mediated massive degranulation which occurs during the immediate hypersensitivity reactions, is typical of a slow degranulation, taking place in delayed hypersensitivity reactions and in chronic inflammatory processes (Kops et al, 1984; Ribatti et al, 1988). In tumours, such as MM, the semilunar aspect of MC secretory granules might correspond to a slow but progressive release of angiogenic factors, in consequence of a chronic and progressive stimulation of MC degranulation.

Tentatively, our data suggest that an increasing number of MCs may be recruited and activated by more malignant plasma cells in active MM, and that angiogenesis in this disease phase may be mediated, at least in part, by angiogenic factors contained in their secretory granules.

### ACKNOWLEDGEMENTS

This work was supported in part by grants from Associazione Italiana per la Ricerca sul Cancro (AIRC, Milan, Italy) to FD and from Ministero dell'Università e della Ricerca Scientifica e Tecnologica (MURST, Funds 60% and ex 40%, Rome, Italy) to DR and AV.

### REFERENCES

Alessandri G, Raju KS and Gullino PM (1984) Characterization of a chemoattractant from endothelium induced by angiogenic effectors. *Cancer Res* **44**: 1579–1584

Aznavoorian S, Murphy AN, Stetler-Stevenson WG and Liotta LA (1993) Molecular aspects of tumor cell invasion and metastasis. *Cancer* **71**: 1368–1383

Beil WJ, Login GR, Galli SJ and Dvorak AM (1994) Ultrastructural immunogold localization of tumor necrosis factor-$\alpha$ on the cytoplasmic granules of rat peritoneal mast cells with rapid microwave fixation. *J Allergy Clin Immunol* **94**: 531–536

Blair RJ, Mengh H, Marchese MJ, Ren S, Schwartz LB, Tonnesen MG and Gruber BL (1997) Human mast cells stimulate vascular tube formation. Tryptase is a novel, potent angiogenic factor. *J Clin Invest* **99**: 2691–2700

© Cancer Research Campaign 1999

Bussolino F, Ziche M, Wang JM, Alessi D, Morbidelli L, Cremona O, Bosia A, Marchisio PC and Mantovani A (1991) *In vitro* and *in vivo* activation of endothelial cells by colony stimulating factors. *J Clin Invest* **87**: 986–995

Castellot JJ, Karnovsky MJ and Spiegelman BM (1982) Differentiation-dependent stimulation of neovascularization and endothelial cell chemotaxis by 3T3 adipocytes. *Proc Natl Acad Sci USA* **79**: 5597–5601

Cozzolino F, Torcia M, Aldinucci D, Rubatelli A, Miliani A, Shaw AR, Lansdorp PM and Di Guglielmo R (1989) Production of interleukin-1 by bone marrow myeloma cells. *Blood* **74**: 380–387

Dethlefsen SM, Matsuura N and Zetter BR (1994) Mast cell accumulation at sites of murine tumor implantation: implications for angiogenesis and tumor metastasis. *Invasion Metastasis* **14**: 395–408

Durie BGM (1991) Staging and kinetics of multiple myeloma. In *Neoplastic Disorders of the Blood*. Wiernik PH, Canellos GP, Kyle RA and Schiffer CA (eds.), pp. 439–451, Churchill Livingstone: New York

Durie BGM and Salmon SE (1977) Multiple myeloma, macroglobulinemia and monoclonal gammapathies. In Recent Advances in Haematology. Hoggbrand AV, Blain MC and Hirsh H (eds), pp. 243–261, Churchill Livingstone: New York

Elias H and Hyde DM (1983) Stereological measurements of isotropic structures. In *A Guide to Practical Stereology*. Elias H and Hyde DM (eds.), pp. 25–44, Karger: Basel

Folkman J (1995) Clinical applications of research on angiogenesis. *N Engl J Med* **333**: 1757–1763

Gruber BL, Marchese MJ and Kew R (1995) Angiogenic factors stimulate mast cell migration. *Blood* **86**: 2488–2493

Grutzkan A, Kruger-Krasagakes S, Kogel H, Schwarz C, Henz BM and Moller A (1996) Synthesis, storage and release of the vascular endothelial growth factor by human mast cells. *Mol Biol Cell* **7**: 352A

Hamada J, Cavanangh PG and Lotan O (1992) Separable growth and migration factors for large-cell lymphoma cells secreted by microvascular endothelial cells derived from target organs for metastasis. *Br J Cancer* **66**: 349–354

Hanahan D and Folkman J (1996) Pattern and emerging mechanisms of the angiogenic switch during tumorigenesis. *Cell* **66**: 353–364

Jakobson AM and Hahnenberger R (1991) Antiangiogenic effect of heparin and other sulphated glycosaminoglycans in the chick embryo chorioallantoic membrane. *Pharmacol Toxicol* **69**: 122–126

Kessler DA, Langer RS, Pless NA and Folkman J (1976) Mast cells and tumor angiogenesis. *Int J Cancer* **18**: 703–709

Klein B (1995) Cytokine, cytokine receptors, transduction signals and oncogenes in human multiple myeloma. *Semin Hematol* **32**: 4–19

Kops SK, Van Loveren H, Rosenstein RW, Ptak W and Askenase PW (1984) Mast cell activation and vascular alterations in immediate hypersensitivity-like reactions induced by a T-cell-derived antigen-binding factor. *Lab Invest* **50**: 421–434

Lichtenstein A, Berenson J, Norman D, Chang M-P and Carlile A (1989) Production of cytokines by bone marrow cells obtained from patients with multiple myeloma. *Blood* **74**: 1266–1273

Meininger EJ and Zetter BR (1992) Mast cells and angiogenesis. *Semin Cancer Biol* **3**: 73–79

Mignatti P and Rifkin DB (1993) Biology and biochemistry of proteinases in tumor invasion. *Physiol Rev* **73**: 161–195

Motro B, Itin A, Sachs L and Keshet E (1990) Pattern of interleukin 6 gene expression *in vivo* suggests a role for this cytokine in angiogenesis. *Proc Natl Acad Sci USA* **87**: 4068–4072

Nakamura M, Merchav S, Carter A, Ernst TJ, Demetri GD, Furukawa Y, Anderson K, Freedman AS and Griffin JD (1989) Expression of a novel 3.5-kb macrophage colony-stimulating factor transcript in human myeloma cells. *J Immunol* **143**: 3543–3547

Norrby K (1993) Heparin and angiogenesis: a low molecular weight fraction inhibits and a high-molecular weight fraction stimulates angiogenesis systematically. *Haemostasis* **23** (suppl. 1): 144–149

Norrby K (1996) Interleukin-8 and *de novo* mammalian angiogenesis. *Cell Proliferation* **29**: 315–323

Norrby K and Whooley D (1993) Role of mast cells in mitogenesis and angiogenesis in normal tissues and tumour tissues. *Adv Biosci* **89**: 71–136

Polverini PF (1996) How the extracellular matrix and macrophages contribute to angiogenesis-dependent diseases. *Eur J Cancer* **32A**: 2430–2437

Poole TJ and Zetter BR (1983) Mast cell chemotaxis to tumor derived factors. *Cancer Res* **43**: 5857–5862

Qu Z, Leibler JM, Powers MR, Galey T, Ahmadi P, Huang XN, Ansel JC, Butterfield JH, Planck SR and Rosenbaum JT (1995) Mast cells are a major source of basic fibroblast growth factor in chronic inflammation and cutaneous hemangiomas. *Am J Pathol* **147**: 564–573

Ribatti D, Roncali L, Nico B and Bertossi M (1987) Effects of exogenous heparin on the vasculogenesis of the chorioallantoic membrane. *Acta Anat* **130**: 257–263

Ribatti D, Contino R and Tursi A (1988) Do mast cells intervene in the vasoproliferative processes of the rheumatoid synovitis? *J Submicrosc Cytol Pathol* **20**: 635–637

Ribatti D, Nico B, Vacca A, Marzullo A, Calvi N, Roncali L and Dammacco F (1998) Do mast cells help to induce angiogenesis in B-cell non-Hodgkin lymphomas? *Br J Cancer* **77**: 1900–1906

Roberts AB, Sporn MB, Assoian RK, Smith JM, Roche NS, Wakefield LM, Heine VI, Liotta LA, Falanga V, Kehr JH and Fauci AS (1986) Transforming growth factor type-beta: rapid induction of fibrosis and angiogenesis *in vivo* and stimulation of collagen formation *in vitro*. *Proc Natl Acad Sci USA* **83**: 4167–4171

Schwab G, Siegall CB, Aarden LA, Neckers LM and Nordan RP (1991) Characterization of an interleukin-6 mediated autocrine growth loop in the human multiple myeloma cell line, U266. *Blood* **77**: 587

Sorbo J, Jakobson A and Norrby K (1994) Mast cell histamine is angiogenic through receptors for histamine 1 and histamine 2. *Int J Exp Pathol* **75**: 43–50

Starkey JR, Crowle PK and Taubenberger S (1988) Mast cell-deficient W/Wv mice exhibit a decreased rate of tumor angiogenesis. *Int J Cancer* **42**: 48–52

Taylor S and Folkman J (1982) Protamine is an inhibitor of angiogenesis. *Nature* **297**: 307–312

Thorton SC, Mueller SM and Levine EM (1983) Human endothelial cells: use of heparin in cloning and long term cultivation. *Science* **222**: 623–625

Vacca A, Ribatti D, Roncali L, Ranieri G, Serio G, Silvestris F and Dammacco F (1994) Bone marrow angiogenesis and progression in multiple myeloma. *Br J Haematol* **87**: 503–508

Vacca A, Moretti S, Ribatti D, Pellegrino A, Pimpinelli N, Bianchi B, Bonifazi E, Ria R, Serio G and Dammacco F (1997) Progression of mycosis fungoides is associated with changes in angiogenesis and expression of the matrix metalloproteinases 2 and 9. *Eur J Cancer* **33**: 1685–1692

Wilks JM, Scott PS, Urla LK and Cocuzza JM (1991) Inhibition of angiogenesis with combination treatments of angiostatic steroids and suramin. *Int J Radiat Biol* **60**: 73–77

*© Cancer Research Campaign 1999*
